# Nonallosteric activation of posttranslational modification enzymes by active site-directed inhibitors

**DOI:** 10.1016/j.csbj.2023.11.019

**Published:** 2023-11-14

**Authors:** Alessandro Pesaresi

**Affiliations:** Istituto di Cristallografia – Consiglio Nazionale delle Ricerche, Area Science Park, Basovizza, Trieste I-34149, Italy

**Keywords:** Activation by inhibition, Exosite, PTM enzymes, Proteases, Protein kinases, Hormesis

## Abstract

Activation-by-inhibition is a biochemical paradox seldom observed in exosite enzymes, wherein active site-bound inhibitors unexpectedly lead to enzyme activation. This intriguing phenomenon occurs at low, undersaturating substrate concentrations, posing a significant challenge in drug discovery, especially when targeting enzymes such as protein kinases, proteases, and other posttranslational modification enzymes. These enzymes often rely on accessory recognition sites known as exosites, which contribute to complex substrate binding mechanisms and unique kinetic behaviors. This study aims to provide a theoretical kinetic explanation for nonallosteric mechanism-based activation-by-inhibition, shedding light on the complexities of inhibiting exosite enzymes solely through active site targeting. Notably, the dual activator-inhibitor behavior of active site-bound inhibitors manifests in a nonmonotonic biphasic dose—response, emphasizing the importance of understanding the role of the inhibitor concentration at low substrate levels. Our findings underscore the potential widespread occurrence of activation by inhibition, a phenomenon that may have been overlooked in the past, and thus advocate for novel strategies in drug design that consider the impact of exosites on enzyme behavior to effectively target exosite enzymes.

## Graphical Abstract

**Figure:**
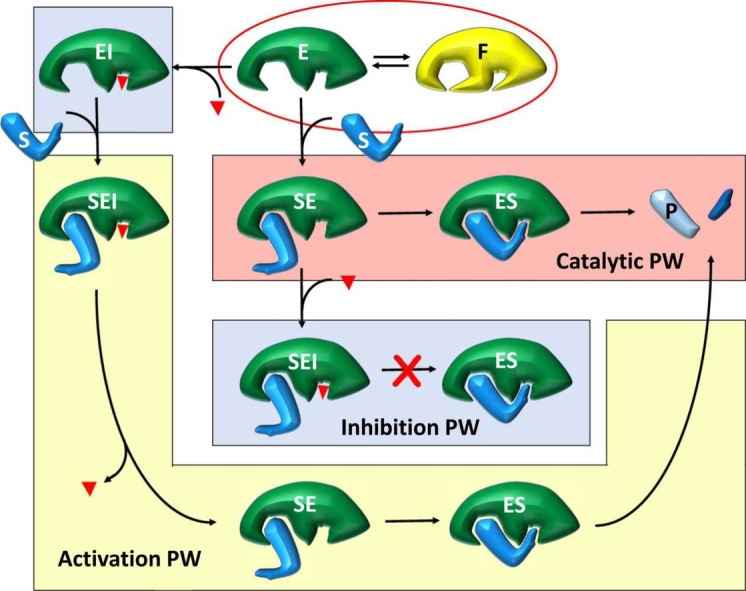

## 1 Introduction

Posttranslational modifications (PTMs) alter protein structure, function and localization, thereby adding huge complexity to proteomes. Catalyzed by a diverse array of posttranslational modification enzymes, more than 700 different types of PTMs have been identified that critically contribute to maintaining cellular homeostasis [1].

Dysregulation of these enzymes has been implicated in a plethora of pathological conditions, including inflammatory diseases, neurodegenerative and cardiovascular disorders, diabetes and cancer [2], [3], [4], [5]. In this complex scenario, proteases and protein kinases stand out as prominent players that orchestrate crucial regulatory mechanisms in many pathophysiologic processes and therefore represent attractive targets for therapeutic intervention in a variety of human diseases [6], [7].

Notably, kinase and protease inhibitors currently account for one-third of all drug discovery research and development efforts. Efforts that, however, are often frustrated by the onset of cardiotoxicity, hypertension, hypothyroidism and other off-target effects that are frequently associated with these inhibitors and that prevent their progress to clinical trials or reduce their therapeutic window [8], [9]. While the precise mechanisms underlying adverse effects remain largely elusive, they are generally attributed to insufficient inhibitor selectivity and subsequent off-target activity [10], [11], [12].

Here, we propose an alternative perspective, suggesting that the high failure rate of drug discovery campaigns targeting proteases and kinases may stem, at least in part, from the paradoxical activation of exosite enzymes triggered by active site-directed competitive inhibitors.

Enzyme activation-by-inhibition is among the most intriguing and counterintuitive behaviors exhibited by enzymes. The crucial characteristic common to all types of linear reversible inhibition is, by definition, the cessation of catalytic turnover: when an inhibitor binds, it hinders the enzyme-substrate interaction in the case of competitive inhibition, or it halts the progress of the catalytic cycle toward the release of the reaction product in the case of uncompetitive inhibition. Either way, at the single-molecule level, enzyme activity comes to a complete standstill. According to conventional theory, this should inevitably lead to a reduction in the reaction rates observed in the bulk. Surprisingly, however, this is not always the case, and over the last decade, numerous examples of enzymes that are nonallosterically activated by active site-bound inhibitors have emerged, challenging traditional assumptions [13], [14], [15], [16], [17], [18], [19], [20], [21], [22].

Attempts to explain the occurrence of this activation-by-inhibition have been made, but they have exclusively revolved around the formal mechanism of mixed inhibition [23], [24], which serves as a useful mathematical tool to model this type of inhibition but does not capture the actual underlying molecular mechanism [25]. Thus, these efforts appear to exploit the mixed-inhibition model rather than providing insights into the plausible mechanisms for activation-by-inhibition.

Interestingly, enzymes that have been reported to undergo activation-by-inhibition all share a common feature: they possess either multiple substrate binding subsites, as observed in β-glucosidases [14] and cholinesterases [22], or exosites that aid in the recognition and stabilization of substrates.

Enzymes that catalyze modifications of large macromolecular substrates, such as endonucleases [26], proteases [27], [28], [29], protein kinases [30], [31], [32], [33], [34] and PTM enzymes in general [35], often derive a significant portion of their substrate affinity from regions of the protein surface that are distinct and remote from the catalytic site, known as exosites. These supplementary recognition sites are believed to confer a level of substrate specificity that cannot be attained solely through the interaction of the consensus sequence with the active site [30], [36], [37], [38], [39], [40].

The study of serine proteases involved in the coagulation cascade provides a compelling example of the interplay between active sites and exosites during substrate recognition. This interaction leads to distinct kinetic behaviors, as illustrated by the cases of active site-bound inhibitors of prothrombinase and intrinsic Xase, resulting in mixed-type inhibition instead of the expected competitive inhibition [41], [42].

Building upon this intriguing observation, we propose a kinetic mechanism that offers a general rationale for the activation of exosite enzymes by active site-bound inhibitors and that might help to explain the occurrence of unexpected side effects caused by inhibitors targeting exosite-containing proteases, protein kinases and other PTM enzymes.

## 2 Results

### 2.1 Conformation-selection model

**Fig. 1:**
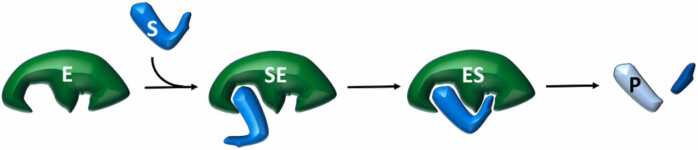
Two-step mechanism of the exosite enzyme-substrate interaction. The large and small pockets on the enzyme (E) represent the exosite and the active site, respectively. S is the substrate, and P is the reaction product(s). Enzyme-substrate recognition is initiated by substrate binding to the exosite with the formation of the encounter complex (SE). SE then undergoes a conformational rearrangement that leads to the catalytic competent complex (ES) where both the exosite and active site are engaged by S. Hence, catalytic conversion takes place, and P is released.

While the actual mechanism of substrate recognition by exosite enzymes can be multifaceted [43], [44], [45], when viewed from a strictly kinetic perspective, it can be reduced down to a two-step process. The first step results in the formation of an encounter complex (SE), where the substrate exclusively binds to the exosite [41], [42]. SE then rearranges to form the catalytically competent complex “ES” (Fig. 1).

**Fig. 2:**
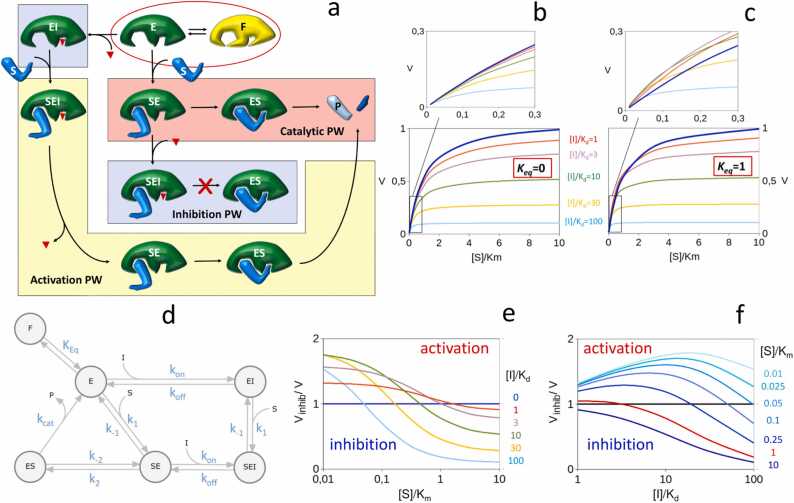
Activation-by-inhibition. (a) Schematic representation of the inhibition and activation mechanism. Free enzyme exists as an equilibrium between active (E) and inactive (F) conformers. Substrate recognition and catalysis are as shown in Fig. 1. The binding of the inhibitor () to the active site of E or SE blocks turnover by hindering the conversion of SE into ES. The dissociation of the inhibitor from the ESI complex allows the rearrangement of SE into ES, enabling catalysis and hence resulting in possible enzyme activation. (b-c) Simulation of exosite enzyme inhibition. Initial reaction velocities plotted as a function of substrate concentrations in the absence (blue traces) or presence of inhibitor concentrations from 1-fold to 100-fold the *K*_*d*_. If *K*_*eq*_= 0 (free enzyme exists only as conformer E), the pattern obtained is typical of mixed-type inhibition (b). If free enzyme exists as an equilibrium between conformers E and F (*K*_*eq*_=1), at low [S], inhibited reactions are faster than uninhibited reactions (c). The insets show a zone of the low substrate saturation region of the plots highlighting the activation-by-inhibition anomaly. (d) Reaction scheme used to simulate exosite enzyme inhibition with KinTek Explorer. (e-f) The inhibition degree, defined as the ratio between inhibited and noninhibited reaction rates, is reported as a function of substrate saturation for different inhibitor concentrations (e) and as a function of inhibitor concentrations for different values of [S]/*K*_*m*_ (f). An inhibition degree less than 1 means inhibition, and a degree higher than 1 means activation.

Based on the idea that enzymes exist as populations of random-fluctuating conformers [46], [47], to explain the occurrence of activation by inhibition, we hypothesize an equilibrium between two forms of the free enzyme, with the active site and exosite that are either accessible by the substrate (conformer E, Fig. 2a) or inaccessible (conformer F). The presence of an E↔F equilibrium diminishes the fraction of the total enzyme that can engage with the substrate. Hence, it reduces the apparent enzyme affinity for the substrate in a competitive inhibition-like fashion. Once the encounter complex (SE) has formed, the turnover proceeds through the formation of the catalytic complex (ES), followed by the catalytic step and the eventual release of the reaction product (Fig. 2a, catalytic pathway).

The inhibitor, however, does not simply cause a dead-end type of inhibition. Since the F, E, SE and EI enzyme forms and complexes are all in equilibrium, the interaction between E and I pushes the E↔F equilibrium away from F and toward E. Under conditions where the rate-limiting step is the E↔F equilibrium (i.e., when [S]≪*K*_*m*_), this alternative path can paradoxically result in enzyme activation (activation pathway, Fig. 2a, and Fig. 2b-c).

Fig. 2d shows the reaction scheme used to simulate this activation-by-inhibitor mechanism using KinTek Explorer [48], [49]. We assumed complete independence between the active site and exosite, making the *on* and *off* rates for substrate and inhibitor binding and unbinding identical for all enzyme forms. This assumption allows us to focus on the critical role of the E↔F equilibrium in the emergence of the inhibition-activation duality.

Provided that enzyme activity is assayed against the proteinaceous physiologic substrate (rather than, for example, against small chromogenic substrates), the binding of an inhibitor to the active site of an exosite enzyme results in an inhibition of the mixed or noncompetitive type [25], [46] (Fig. 2b). The path E→EI→SEI→SE (Fig. 2d), by enabling an alternative route to the catalytic turnover, reduces the inhibitory effect independently from the E↔F equilibrium.

For the enzyme modeled in our theoretical example, if the E-F equilibrium constant (*K*_*eq*_) is set to 0 (which implies that the free enzyme exists solely as the E conformer), the steady-state analysis of an inhibitor with a *K*_*d*_ of 1 µM gives an observed competitive inhibition constant (*K*_*ic*_) of 104 µM. Thus, a first notable singularity of exosite enzymes is that they tend to neutralize the inhibitory effect of active site-directed ligands, turning, as in this case, relatively strong binders into poor inhibitors.

To understand the underlying mechanism of activation-by-inhibition, it is convenient to first focus on the inhibitory behavior observed in the absence of an E↔F equilibrium and under the assumption of active and exosite independence. When the equilibrium constant *K*_*eq*_= 0, the free enzyme E is contended only by S and I. Since the E + S→SE and EI+S→SEI steps are under any kinetic aspect equivalent, it is evident that, regardless of the specific combination of [S] and [I], the net rate through the E→EI→SEI→SE path is always necessarily slower than the rate of the E→SE step. Consequently, although the inhibitory pathway partially contributes to the turnover rate, the binding of the inhibitor inevitably leads to an overall reduction in enzyme activity.

However, when an equilibrium is established between conformers E and F (*K*_*eq*_=1 in our example) and the substrate concentration is below saturation, the inhibition pattern becomes tangled up by the occurrence of activation-by-inhibition (Fig. 2**c**). This phenomenon occurs because the free enzyme E is now engaged in a triple equilibrium, and the binding of the inhibitor recruits enzyme molecules not only from the E pool through the E↔EI and SE↔E↔EI equilibriums, as in the previous case but also from the F pool through the F↔E↔EI equilibrium.

The diversion of E and SE toward EI still contributes to a decrease in the turnover rate because the inhibition/activation pathway is inherently slower than the catalytic pathway. However, under conditions of substrate undersaturation, when [S]⪅*K*_*m*_ and [I]⪆*K*_*d*_, the activity loss resulting from E↔EI and SE↔E↔EI diversion can be exceeded by the gain enabled by the recruitment of the F pool. Consequently, the activation-by-inhibition anomaly emerges.

From a kinetic perspective, it is intriguing to observe that the binding of the inhibitor to the enzyme’s active site gives rise to a paradoxical increase in the apparent affinity for the substrate (Fig. 2c). As the substrate concentration approaches saturation, the competitive-like effect exerted by the E↔F equilibrium fades off, and the activation path transitions into the inhibitory mode (Fig. 2e).

Notably, the activation exhibits a nonmonotonic dependence on the inhibitor concentration: an increase in [I] shifts the E↔EI equilibrium toward EI, thereby increasing the flux through the activation pathway. However, it also promotes the backward reaction of SE toward the SEI, intensifying the inhibitory nature of this path. Consequently, when [S] is far from saturation, an increase in [I] can lead to either an increase or a reduction in enzyme activity (Fig. 2f).

In summary, the interplay of these three mechanisms – the competition of the E↔F equilibrium with the catalytic pathway, the recruitment of the F pool by the inhibitor and the nonmonotonic dependence of the activation pathway on [I] – results in an intricate inhibition/activation pattern that is triggered by [S]/*K*_*m*_ and [I]/*K*_*i*_, as shown in Fig. 2e and f.

While an analytical description of this mechanism would be overly complex and of limited practical utility, it is worth noting that, under the assumption of active and exosite independence, the inhibition-to-activation transition critically requires the presence of an E↔F equilibrium. Moreover, the onset of activation is contingent on both the dissociation rate of the inhibitor (EI→E + I) and the dissociation of S from the ternary SEI complex (SEI→EI+S). It is indeed the fate of SEI that governs the possibility of activation-by-inhibition. Hence, irrespective of the EI dissociation rate, as long as *k*_*-1*(SEI)_ is lower than *k*_*off*_, activation-by-inhibition remains feasible, provided that [S] remains sufficiently low. The extent of maximum activation observed at a given [S] increases with greater *K*_*eq*_ and a higher *k*_*-1*(SEI)_/ *k*_*off*_ ratio.

### 2.2 Activation/inhibition dependence on F conformer substrate/inhibitor accessibility

**Fig. 3:**
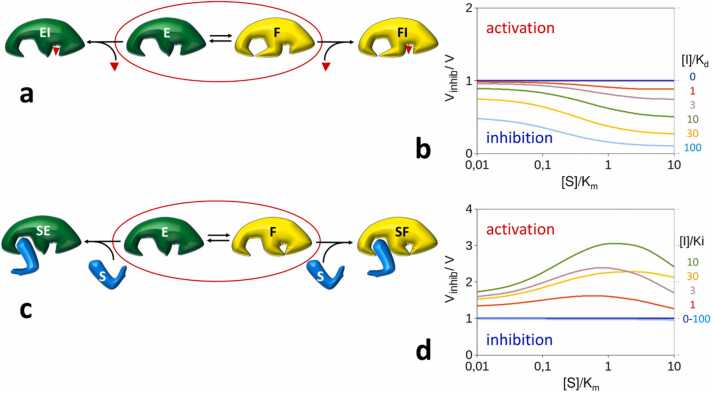
Structure of the F conformer and constitutive activation/inhibition. If in the F inactive conformer the active site remains properly formed and accessible by the inhibitor, the F-I interaction must be considered (**a**). In this case, under the assumption of active and exosite independence, the enzyme-inhibitor interaction does not perturb the E↔F equilibrium. Consequently, there is no recruitment of the F pool, and activation-by-inhibition does not take place (b). In cases where the active site in the F conformer is inaccessible to the inhibitor, while the exosite remains open to the substrate (**c**), the E↔F equilibrium is insensitive to [S]; hence, active-site binders result in constitutive activation-by-inhibition (**d**).

Thus far, our discussion has focused exclusively on scenarios where the inactivity of the F conformer results from the unavailability of both the active site and exosite. However, it is quite conceivable that inactivity is determined by the inaccessibility of just one of these two sites. Specifically, if in the F conformer the active site is properly formed while the exosite is inaccessible, we must address the F↔FI equilibrium, as depicted in Fig. 3**a**. Under these circumstances, the binding of I only leads to inhibition, and no activation can occur (Fig. 3b). This is because, assuming active and exosite independence, the inhibitor binds with equal strength to the active site of E and F, which, in turn, has no impact on the E↔F equilibrium, precluding any recruitment of the F pool.

Of more significant interest is the scenario where the active site undergoes transitions between open and closed conformations, while the exosite remains accessible. This situation is frequently observed in proteases, where to prevent indiscriminate proteolysis, in the fundamental free enzyme state, the active site is not properly formed or is not accessible. Hence, the inhibitor cannot bind to the F conformer, but the substrate can still bind the exosite (Fig. 3**c**). In this case, it is the binding of S that has no impact on the E↔F equilibrium, resulting in a sort of noncompetitive-like inhibition that causes a significant increase of the apparent *K*_*m*_. Unlike the scenario described in section 3.1, in this case even at high [S], the substrate is unable to shift the E↔F equilibrium toward E, causing the E + I→EI binding to exert a quasi-constitutive enzyme activation that is observed even at [S]≫*K*_*m*_ (Fig. 3d).

### 2.3 Generalization of the model with extended applicability

**Fig. 4:**
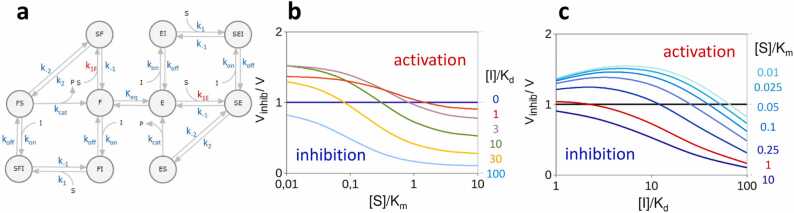
Extended model of exosite enzyme activation by inhibition. (a) Reaction scheme. Conformers E and F are both active and exhibit identical kinetic properties except for the substrate binding rate (*k*_*1(F)*_*= 10·k*_*1(E*_). (b) Inhibition degree as a function of substrate saturation at different [I]/*K*_*d*_ values and as a function of [I]/*K*_*d*_ at different substrate concentrations (c).

To simplify the description of the activation model, we have assumed that the F conformer is completely inactive. However, it is important to recognize that such a strict requirement is not necessary for activation-by-inhibition to take place. Fig. 4 demonstrates an alternative scenario, where both the E and F conformers are active, sharing identical kinetic properties, except for a 10-fold difference in the microscopic rate of substrate binding to the exosite (i.e., the F conformer has a 10-fold higher *K*_*m*_). Here, *K*_*eq*_ was again set to 1. Because of the competition between the E↔F and E↔SE steps, if *K*_*eq*_ is allowed to assume larger values, a sharp activation-by-inhibition anomaly occurs even if the *K*_*m*_ of the F conformer is only slightly higher than that of E. This example demonstrates that activation-by-inhibition can arise whenever different forms of the free exosite enzyme are in thermodynamic equilibrium and possess varying affinities for the substrate. Therefore, this mechanism holds general validity and extends its applicability beyond the specific assumption made in the simplified model.

### 2.4 The biphasic dose—response curve and a case study

In operational terms, the key distinction between allosteric and nonallosteric activation-by-inhibition lies in the substrate and inhibitor concentrations needed to trigger the activation process. In cases driven by allostery, such as the protease ClpP, the heat shock protease DegP or the human protease HTRA1, the binding of the inhibitor triggers structural modifications that alter the kinetic properties of the enzyme [50], [51], [52]. In these cases, activation strictly requires low substoichiometric inhibitor concentrations and, most notably, is independent of the substrate concentration. While in our nonallosteric mechanism-based model, activation is only observed at low substrate, it is induced by inhibitor concentrations within the *K*_*d*_ range or higher and leads to the paradoxical increase in apparent substrate affinity.

**Fig. 5:**
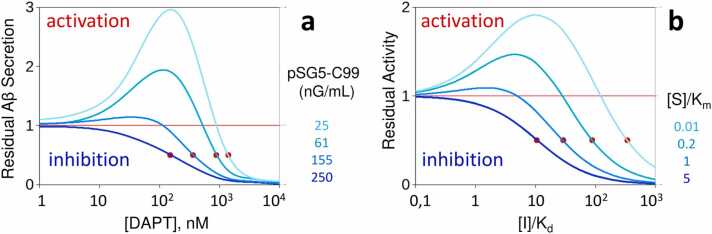
Dose—response curves. γ-Secretase inhibition by DAPT (**a**). Aβ-peptide residual secretion by HeLa cells transfected with increasing concentrations of the pSG5-C99APP expression vector is reported as a function of the DAPT inhibitor concentration. When the C99-APP substrate is expressed at high levels, DAPT dosing produces the typical dose—response curve (dark blue trace). As substrate concentration decreases, DAPT induces enzyme activation if [DAPT] ⪅ 10^2^ nM and enzyme inhibition if [DAPT] ⪆ 10^2^ nM, resulting in a biphasic bell-shaped curve. The inhibitor IC50, indicated by red dots, exhibits a marked dependence on substrate concentration, increasing from 150 nM (at high substrate levels) to 1.4 µM (at low substrate concentrations). (**b**) Dose—response curves generated by our model, implementing the mechanism of Fig. 2d (with *K*_*eq*_=1.5).

The literature offers several instances of enzymes whose activation-by-inhibition conforms to our nonallosteric model. Among the four most prominent examples, we can list the inhibitor-induced activation of NAD-dependent histone deacetylase sirtuins [17], c-Src kinase activation by dasatinib [18] and PERK and GCN2 kinase activation by ATP-competitive inhibitors [19]. Both in cell-based assays and in vitro investigations employing purified enzymes, inhibition of these enzymes has consistently been reported to result in nonmonotonic, bell-shaped dose responses that arise from the interplay of substrate and inhibitor concentrations. Remarkably, the nature and structure of the biphasic activation/inhibition curves replicate the behavior exhibited by our theoretical mechanism, as illustrated in Fig. 5. Unfortunately, these studies lack in-depth kinetic characterizations of the activation mechanism. Therefore, it remains challenging at present to accurately fit our model to the available data. A partial exception is the membrane-embedded protease γ-secretase, for which a sufficiently broad range of substrate and inhibitor concentrations have been explored, allowing a qualitative comparison between our theoretical model and experimental observations.

Data were adapted from reference [15].

γ-Secretase is a transmembrane protein complex comprised of four essential components: NCT, APH-1, PEN-2, and the catalytic subunit presenilin. This enzyme belongs to a family of intramembrane-cleaving proteases responsible for controlled proteolysis of transmembrane peptides and generation of biologically active signaling fragments. Notably, γ-secretase plays a critical role in the pathogenesis of Alzheimer’s disease through its implication in the processing of the β-amyloid precursor protein (APP). The initial step in this process involves the extracellular cleavage of APP by BACE, resulting in the generation of a C-terminal 99-residue fragment known as C99-APP. Subsequently, γ-secretase further processes C99-APP, yielding various lengths of Aβ peptides. The accumulation of these Aβ peptides is closely associated with the formation of β-amyloid plaques, a hallmark feature of Alzheimer's disease. Over the past three decades, extensive endeavors have been dedicated to the design of γ-secretase inhibitors aimed at the development of medications that could alter the progression of Alzheimer's disease. Regrettably, these initiatives have resulted in repeated failures, as illustrated by the notable examples of presenelin inhibitors semagacestat and avagacestat, whose clinical trials were prematurely halted due to their association with accelerated cognitive decline and exacerbation of Alzheimer's symptoms [53], [54].

In accordance with these findings, numerous studies have consistently documented that γ-secretase inhibitors can elevate Aβ production. This Aβ-rise, observed in rat brain, cell-based assays and experiments with purified solubilized enzyme, has been demonstrated to manifest only at low enough concentrations of the C99-APP substrate [55]. Plotted as a function of inhibitor concentration, the inhibited γ-secretase activity exhibits a bell-shaped dose—response curve [15], [16], (Fig. 5a), displaying striking resemblance to the activity-versus-inhibitor concentration pattern generated by our theoretical model (Fig. 5b).

Deeper insights drawn from the structural and mechanistic characteristics of γ-secretase further underscore the parallels between our activation-by-inhibition model and this specific case. For instance, recognition between γ-secretase and C99-APP involves an exosite located between the NCT and PEN-2 subunits. The substrate is then translocated to the active site via an intermediate second exosite situated between the NCT and PS subunits [56]. Other indirect evidence supporting the formation of a transient encounter complex between C99-APP and γ-secretase comes from the discovery that active site-directed inhibitors such as PME and L685458 cause noncompetitive inhibition [16], [57], as opposed to what one might expect from competitive active site binders. This is reminiscent of the noncompetitive inhibition reported by Krishnaswamy in the case of active-site inhibitors of exosite serine proteases within the blood coagulation cascade [28], [41], [42]. Moreover, substrate access to the active site is expected to involve movements of transmembrane domains 2 and 6 of presenilin, which display substantial flexibility in the atomic structure of the enzyme. This view is also supported by structural data showing that the flexibility of domain 2 is greatly reduced upon binding of DAPT inhibitors [56]. This suggests that the unbound form of the presenilin protease exists as a combination of conformers where the active site is either accessible or inaccessible to the substrate and that the formation of the catalytic competent ES complex follows a conformational selection mechanism [58].

## 3 Methods

The activity of a prototypical exosite enzyme, adhering to the mechanism expounded in the Results Section 2.1 and following the reaction schemes illustrated in Figs. 2d and 4a, was simulated using KinTek Explorer [48], [49]. For the simulation of enzyme inhibition/activation, the microscopic rate constants were as follows:

*k*_*1*_= 10^7^ mol^−1^s^−1^, *k*_*-1*_= 10 s^−1^, *k*_*2*_= 10^3^ mol^−1^s^−1^, *k*_*-2*_= 1 s^−1^, *k*_*cat*_= 100 s^−1^, which gives *K*_*m*_= 10 µM. *k*_*on*_= 10^9^ mol^−1^s^−1^, *k*_*off*_= 10^3^ s^−1^, corresponding to a *K*_*d*_ of 1 µM. The equilibrium constant *K*_*eq*_ is defined as the ratio between the forward E→F conversion and the backward E←F conversion (*k*_*fwd*_= *k*_*bkwd*_=10^3^ s^−1^).

The enzyme concentration was 100 pM, the substrate concentrations were between 0.1 and 100 µM, and the inhibitor concentrations were between 0 and 100 µM.

The computed initial reaction velocities have been reported as a function of substrate concentrations (expressed as [S]/*K*_*m*_) and inhibitor concentrations (expressed as [I]/*K*_*d*_).

## 4 Discussion

To what extent might the nonallosteric activation mechanism proposed herein be significant in a real-life cellular context? To address this question, we must concentrate on the three preconditions necessary for the onset of the activation-by-inhibition anomaly:

i. two-step enzyme-substrate recognition mechanism with the formation of an intermediate encounter complex
ii. thermodynamic equilibrium between native enzyme conformers with differential substrate affinities
iii. undersaturating substrate concentrations

The serine proteases involved in the blood coagulation cascade represent exemplary instances of exosite enzymes, and an extensive body of literature delves into the intricate regulation of thrombin, along with the central role played by its exosites (reviewed in reference [59]). The two-step enzyme-substrate binding scheme incorporated in our activation model draws inspiration from the mechanism of prethrombin 2 cleavage by prothrombinase and the cleavage of factor X by the Xase complex, as elucidated by Krishnaswamy *et al*. [28], [41], [42].

While it is reasonable to expect variations in the actual mechanism of interaction between exosite enzymes and substrates, certain common features can be anticipated. For instance, assuming that the majority of docking energy originates from exosite interaction implies that binding must commence with substrate-exosite recognition, hence leading to the formation of a transient encounter complex [30], [37], [39]. This highlights the potential of the two-step mechanism as a valuable paradigm for substrate recognition by exosite enzymes in general.

The second requirement, namely, the existence of an equilibrium between two enzyme conformers with different substrate affinities, may seem to be a somewhat arbitrary condition and is perhaps the primary objection to the widespread applicability of the activation-by-inhibition model. However, it must be considered that the role played by PTM enzymes in the regulation of metabolism, the cell cycle and other essential processes requires that they respond to diverse cellular and environmental stimuli [60], [61]. To meet the demands of these complex regulations, protein kinases and proteases, for example, often adopt flexible multidomain architectures, and the recognition of their substrates frequently entails a conformation-selection mechanism between active and inactive conformers [62], [63]. An illustration of this conformational regulation is frequently observed in native proteases, which are inactive to prevent indiscriminate proteolysis and become active only upon interaction with their substrates. Consequently, the presence of an equilibrium between native enzyme conformers with distinct substrate affinities in proteases, protein kinases and PTM enzymes in general is not only plausible but is likely more common than in average metabolic enzymes.

The third precondition, enzyme undersaturation, merits a more in-depth discussion, as it might play a crucial role in the discrepancy often observed between the outcomes of preclinical and clinical studies on protease and protein kinase inhibitors.

Although accurate data on cellular metabolite concentrations are rarely available, both theoretical considerations regarding the regulation of metabolic fluxes [64], [65] and experimental evidence [66], [67], [68], [69] suggest that within cells enzymes generally function in conditions far from substrate saturation. Specifically, this appears to be the norm for proteases. For instance, circulating levels of coagulation cascade enzymes are typically in the nM range [70], while the *K*_*m*_ of thrombin-catalyzed conversion of human thrombinogen is in the µM range [71], and the *K*_*m*_ of factor X cleavage by intrinsic Xase is approximately 500 µM [72]. Similarly, γ-secretase, which is involved in the generation of the Aβ peptide in the brain of Alzheimer’s disease patients, operates physiologically in a regime of high undersaturation [55], [73]. Other examples of proteases with low or very low substrate affinity that exploit low saturation conditions include the membrane rhomboid protease, which has a *K*_*d*_ for its substrate of ∼200 µM [74], and matrix metalloproteinases, whose *K*_*m*_ is in the 5–20 µM range [75].

Substrate undersaturation seems to be a common condition for protein kinases as well. Several lines of research have highlighted that the optimal efficacy of signal transduction, which occurs through the phosphorylation cascades catalyzed by protein kinases, is achieved when the kinase substrates are at concentrations significantly below saturation [76], [77], [78]. This observation aligns with the finding that protein kinases often display a rather low affinity for their proteinaceous substrates, typically in the high µM range [79], [80], [81], strongly suggesting that these enzymes also function under large undersaturation in physiological settings.

Overall, this explains why experimental evidence of activation-by-inhibition has not been reported more frequently: to detect activation, the inhibition study must be performed using the physiologic protein substrate, which is rarely done due to cost and complexity. Furthermore, even when the proteinaceous substrate is used, activation can only be observed when the substrate concentration is far below saturation. Inhibition assays, on the contrary, usually employ far more convenient chromogenic small-molecule substrates and are optimized to enhance the detection of a dose—response effect, prioritizing high substrate concentrations over replicating physiological conditions and hence inadvertently overlooking potential activation-by-inhibition [16].

In addition to offering a general explanation for the occurrence of activation-by-inhibition, the kinetic model we propose also provides a mechanistic rationale for the occurrence of hormesis [82], a biphasic dose—response mechanism characterized by low-dose stimulation and high-dose inhibition. Hormetic dose responses have increasingly gained prominence as toxicologists and pharmacologists intensify their investigations into potential responses within the low-dose range [83].

Noteworthy examples of hormesis in pharmacology have been reported for various agents, including anticancer drugs [84], [85], [86], endocrine modulators [87], antiangiogenic agents [88], [89], and protease inhibitors [13], [73].

Although the precise mechanistic determinants of hormesis often remain unknown, these nonmonotonic biphasic dose responses are generally recognized as adaptive responses [90] and are expected to occur through intricate multicomponent physiologic reactions, encompassing the modulation of signaling pathways and gene activity [91], [92], [93].

Indeed, our model reveals that the interaction of competitive inhibitors with the active site of exosite enzymes can potentially lead to nonmonotonic inhibitory responses (Fig. 2f). This intriguing observation sheds light on the possibility that hormesis, at least in certain instances, could arise from the inhibition of single exosite enzymes rather than from complex physiologic adaptations, adding a new dimension to our understanding of enzymatic regulation.

In conclusion, this paper introduces a theoretical model proposing nonallosteric activation of exosite enzymes by active site inhibitors. Although experimental validation for our specific model is yet to be provided, there is substantial alignment between our simulations and the experimentally observed inhibitor-induced activation of several PTM enzymes, including histone deacetylase [17], the kinases c-Src [18], PERK and GCN2 [19], and, most notably, γ-secretase [15], [16].

It is important to specify that several deviations from our model can be expected to prevent the onset of nonallosteric activation-by-inhibition. This can be due to various factors, such as the absence of exosites in some PTM enzymes, the constitutive activity of the unbound enzyme with no transition between active and inactive conformers, or rapid conversion of the encounter complex (SE) into the catalytic complex (ES), making the SE→ES step kinetically irrelevant. Moreover, allosteric cross-talk between the active site and exosite can either accentuate or abolish the onset of activation. In some circumstances, the physiologic substrate concentration might fall within or exceed the *K*_*m*_ range. Additionally, for high potency inhibitors, EI dissociation might be slower than SEI→EI+S dissociation. Hence, it is evident that activation-by-inhibition should not be regarded as a phenomenon universally applicable to all PTM enzymes.

Nevertheless, this study illuminates unexplored potential in enzyme behavior and suggests that should this activation mechanism be actively searched for, the prevalence of PTM enzymes that are activated by inhibitors might significantly increase. In addition, our theoretical model offers a conceptual framework that can assist in making sense of seemingly paradoxical evidence and may stimulate targeted research endeavors. Investigating a wide range of [S]/*K*_*m*_ and [I]/K_i_ combinations on PTM enzyme activity can enhance our comprehension of the conditions needed for effective inhibition, both in vitro and in vivo, of vital pharmacological targets. These insights may contribute to optimizing resources in drug research, preventing wasteful efforts on hits and lead compounds that may never progress to approved medications.

## Author statement

As the only author of the present paper, Alessandro Pesaresi has taken care of all aspects of this work.

## Declaration of Competing Interest

Authors declare no conflict of interests.
